# Electronically Switchable Sham Transcranial Magnetic Stimulation (TMS) System

**DOI:** 10.1371/journal.pone.0001923

**Published:** 2008-04-09

**Authors:** Fumiko Hoeft, Daw-An Wu, Arvel Hernandez, Gary H. Glover, Shinsuke Shimojo

**Affiliations:** 1 Center for Interdisciplinary Brain Sciences Research (CIBSR), Stanford University School of Medicine, Palo Alto, California, United States of America; 2 Computation and Neural Systems and Division of Biology, California Institute of Technology, Pasadena, California, United States of America; 3 Department of Psychology, Harvard University, Cambridge, Massachusetts, United States of America; 4 Department of Radiology, Stanford University School of Medicine, Palo Alto, California, United States of America; Harvard Medical School, United States of America

## Abstract

Transcranial magnetic stimulation (TMS) is increasingly being used to demonstrate the causal links between brain and behavior in humans. Further, extensive clinical trials are being conducted to investigate the therapeutic role of TMS in disorders such as depression. Because TMS causes strong peripheral effects such as auditory clicks and muscle twitches, experimental artifacts such as subject bias and placebo effect are clear concerns. Several sham TMS methods have been developed, but none of the techniques allows one to intermix real and sham TMS on a trial-by-trial basis in a double-blind manner. We have developed an attachment that allows fast, automated switching between *Standard* TMS and two types of control TMS (*Sham* and *Reverse*) without movement of the coil or reconfiguration of the setup. We validate the setup by performing mathematical modeling, search-coil and physiological measurements. To see if the stimulus conditions can be blinded, we conduct perceptual discrimination and sensory perception studies. We verify that the physical properties of the stimulus are appropriate, and that successive stimuli do not contaminate each other. We find that the threshold for motor activation is significantly higher for *Reversed* than for *Standard* stimulation, and that *Sham* stimulation entirely fails to activate muscle potentials. Subjects and experimenters perform poorly at discriminating between *Sham* and *Standard* TMS with a figure-of-eight coil, and between *Reverse* and *Standard* TMS with a circular coil. Our results raise the possibility of utilizing this technique for a wide range of applications.

## Introduction

Transcranial magnetic stimulation (TMS) is an increasingly popular neuroscience tool due to its unique ability to noninvasively alter neural activity in targeted regions of the brain [1]. Since its introduction in 1985 by Barker and colleagues [2], TMS has been used to probe motor cortex excitability [3]–[6], map motor and cognitive functions [7], [8], study anatomical and functional connectivity [8], [9], and modulate brain function with therapeutic aims [6], [10], [11].

TMS uses a time-varying magnetic field to induce an electrical current through the skull, in a spatially restricted region of the cerebral cortex. The induction of electrical current occurs with minimal attenuation of the magnetic field. Significant currents can be induced without having to apply substantial voltages across the skull, minimizing the activation of pain fibers and pain sensation. The advantage of TMS is also in its temporal (sub-millisecond) and spatial (sub-centimeter) resolution.

Two configurations of TMS coils are commonly used in scientific and clinical research. The figure-of-eight coil (also known as butterfly or double coils) is the most commonly used configuration owing to its superior spatial specificity. The circular coil is less used because while it offers more powerful stimulation and the opportunity to target both motor cortices at the same time with relatively little worry about specific placement or constant positioning, it is also less focused. It has been used in clinical trials that targets large regions of the brain, such as investigations of Parkinson's disease and epilepsy [12] and motor physiology studies [13]. Its specificity can also be improved when applied to brain regions where the preferred current direction is known, such as the motor and visual cortices [13]–[15].

As with any experimental technique, TMS has its pitfalls [16]. Specifically, TMS is accompanied by a number of ancillary effects. The coil emits clicking sounds with each stimulation, and can also stimulate nearby peripheral nerves and muscles. Depending on the location and strength of TMS, this may result in sensations ranging from a light tapping on the scalp to uncomfortable muscle twitches in the face, neck, or shoulders. These sensations can nonspecifically interfere with task performance via distraction or subject biasing, contaminating the results. In clinical research, placebo effects are known to be high [17], [18], especially with medical devices where there is significant patient-investigator contact [19].

To separate the effects of brain stimulation from those arising from the above artifacts, experimenters can compare results with control conditions in which they either apply sham stimulation or apply real stimulation to a control brain region. These two methods are complementary to one another; one may not be necessary in some studies, and in other studies, stimulation of control brain regions methods may still be necessary in addition to sham TMS (to show specificity of the brain region of interest). Ideally, the experimental and control conditions should differ only by the way in which brain is stimulated, while producing auditory and tactile artifacts that are not easily distinguishable from real stimulation. See **Supporting Information Text S1** for detailed discussion about different types of control (including sham) conditions that are available. Furthermore, the conditions should be easily interleaved to allow within-subject comparisons and intermix various conditions trial-by-trial.

The goal of this study was to develop and fully validate a method of delivering several control TMS conditions. Two coils were fabricated; a figure-of-eight coil (Fig8) that has loops of coils in each of the two wings that are driven separately, and a circular coil (Circ) that has two sets of coils stacked on top of another that are also driven separately. An attachment allows the delivery of three types of stimuli in an automated, interleaved manner without switching or moving the coil (single-trial sham TMS). 1) Standard stimuli are delivered when current direction in both loops matches that of the standard coils. 2) Sham stimuli are delivered when current direction in one of the two loops is backwards. 3) Reversed stimuli are delivered when current direction in both loops is backwards. Reversed stimuli reproduce the fields created by coil-flipping, which can be used to increase activation thresholds over brain areas where the preferred stimulus orientation is known, such as motor [20]–[23], visual [24] and prefrontal cortices [25]. In the case of motor and visual areas, these can also be used to preferentially stimulate either hemisphere from a single coil location.

We extend upon Ruohonen et al.'s design of a sham Fig8 coil [26]. We add independent control of current direction in *both* coil loops so that reverse stimulation is possible in addition to sham and standard stimulation. Further, automated electronic switching of stimulus types can be done within 3 ms with a solid state switch known as thyristors, and we apply the design to both Fig8 and Circ coils. In addition, one can adjust stimulation intensity of each current to achieve complete cancellation of the induced fields (with circular coils, since there is some distance between the two loops of coils, the stimulus intensity necessary to achieve complete cancellation for each loop is different). To enhance the applicability of the design, we implement it in an attachment to Magstim single- and dual-pulse setups, which are in common use in research and clinical settings.

Four types of experiments were performed to validate the Standard, Reversed and Sham TMS delivered from the Fig8 and Circ coils. First, in order to characterize physical properties of the stimuli such as electro-motive force (EMF), we performed mathematical modeling and actual measurements using a search-coil. This included both measurements of single pulses and of successive pulses to ensure that stimulus properties were not contaminated by prior stimuli via residual states in the circuitry. Second, we measured the physiological effects of the stimulus types by comparing thresholds for eliciting motor evoked-potentials (MEPs) when stimulating primary motor cortex. Third, we tested the perceptual effects of the different pulses by testing whether subjects and experienced investigators could differentiate Sham stimuli, and if so whether Standard and Reversed could be differentiated (which may serve as another form of sham TMS). Finally, sound pressure level (SPL), subjective loudness and pain intensity were measured to further characterize their effects on the subjects.

## Results

### Mathematical Modeling of Electro-Motive Force (EMF)

**Figure 1:**
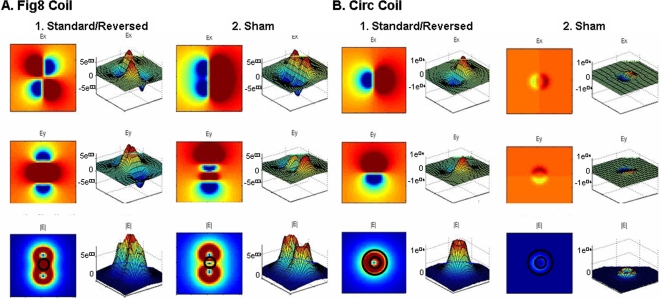
Modeled Electric fields of Standard and Sham TMS with Fig8 (A) and Circ (B) coils. Electric field for x (top panel) and y (middle panel) axes and strength (bottom panel) are plotted as both color and height on the 3-d images and sas color on the 2-d images. Black circles indicate approximate locations of peaks when commercially available coils are used (Fig8: within annulus, Circ: between the two circles).

Using simulations, we modeled electric fields for Standard, Reversed and Sham TMS for both the custom-made Fig8 and Circ coils. The induced electric field strength is thought to be one critical parameter determining the excitation of cortical tissue [27], [28]. Reversed TMS was not modeled, as the only difference between Standard and Reversed TMS for either coil was the direction of the field. The model results for Standard TMS (Fig. 1) are consistent with measurements of commercially available coils showing peak electric fields at the intersection between the two wings of coils for the Fig8 coil and along the circumference for the Circ coil.

When Sham TMS is applied through the Fig8 coil, the central peak is eliminated, but the smaller surrounding peaks remain similar in absolute magnitude. In the Circ coil, the fields are uniformly and drastically diminished in strength.

### Search Coil Measurements of EMF

In the second series of experiments, we measured EMF (proportional to the current which would be induced in the tissue) of various TMS pulses applied to a search coil. First we compared EMF amplitude between Standard, Reversed and Sham TMS through the Fig8 and Circ coils using independent t-tests. There were no significant differences in EMF between Standard and Reversed (Fig8: t_(38)_ = 0.12, p = 0.91; Circ: t_(38)_ = 0.17, p = 0.87). There were however, significant differences in EMF between Standard (or Reversed) and Sham (Fig8: t_(38)_ = 29.2, p<0.001; Circ: t_(38)_ = 15.5, p<0.001).

**Figure 2:**
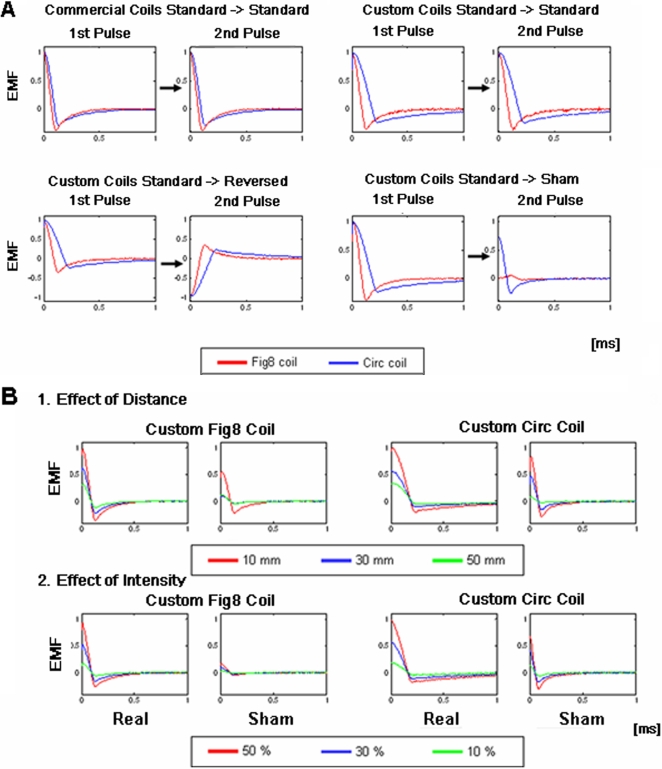
Electromagnetic force (EMF) measurements of Standard, Reversed and Sham TMS with Fig8 and Circ coils. (A) Effects of 1^st^ pulse on 2^nd^ pulse. Two consecutive pulses were delivered at 10 ms interstimulus interval (ISI) at 50% maximum output and EMF was measured. All EMF values are normalized to the 1^st^ pulse. (B) Effects of distance, intensity and position. B-1. EMF measured at varying distances between custom Fig8 or Circ coils and search coil. Values are normalized to those of Standard TMS at 10 mm. B-2. EMF measured at varying TMS intensity with custom Fig8 and Circ coils. Values are normalized to those of Standard TMS at 50% maximal output. For clarity, all plots show data for Reversed pulses as inverted and collapsed with data for Standard pulses, as they showed no significant differences beside their polarities.

Next, we compared the EMF amplitudes of two TMS pulses delivered at an ISI of 10 ms to investigate whether there were any residual effects in the electronics that would cause contamination of the second pulse at this short inter-trial interval (ISI). When we examined EMF induced by commercially available Fig8 and Circ coils, we found no effect of the 1^st^ pulse on the 2^nd^ pulse, i.e., there were no significant differences between the 1^st^ and 2^nd^ EMF (Fig8: t_(38)_ = 0.03, p = 0.98; Circ: t_(38)_ = 0.10, p = 0.92; Fig. 2A top left panel). When two consecutive Standard (or Reversed) TMS were delivered using custom-made coils, both Fig8 and Circ coils also showed no significant differences in EMF (Fig8: t_(38)_ = 0.10, p = 0.92; Circ: t_(38)_ = 0.23, p = 0.82; Fig. 2A top right panel). When Reversed was delivered after Standard TMS (or Standard after Reversed), similarly there was no significant effect of the 1^st^ pulse on the 2^nd^ (Fig8: t_(38)_ = 0.14, p = 0.89; Circ: t_(38)_ = 0.02, p = 0.97; Fig. 2A bottom left panel). Finally, we tested the effect of Standard or Reversed TMS (1^st^ pulse) on Sham TMS (2^nd^ pulse). There were no significant differences between EMF of single-pulse Sham TMS and the 2^nd^ pulse Sham TMS (Fig8: t_(38)_ = 0.39, p = 0.70; Circ: t_(38)_ = 0.28, p = 0.78; Fig. 2A bottom right panel). In sum, no significant interactions were found in any of the combinations tested.

We then measured the decay of stimulation with increased distance by placing the search coil at distances from 10 to 50 mm away from the custom-made Fig8 and Circ coils. There was a monotonic decrease in EMF for Standard (and Reversed) and Sham TMS as the distance increased (Fig. 2B-1). For both the Fig8 and Circ coils, EMF amplitude measures using one-way repeated measures analysis of variance (ANOVA) showed significant main effects of distance (10 30, 50 mm) for all TMS type (Standard/Reversed, Sham) and coils (Fig8, Circ) (Fig8-Standard/Reversed: F_(2, 57)_ = 104.2, p<0.001; Fig8-Sham: F_(2, 57)_ = 1139.5, p<0.001; Circ-Standard/Reversed: F_(2, 57)_ = 22510.0, p<0.001; Circ-Sham: F_(2, 57)_ = 7441.6, p<0.001).

In addition, we measured the effect of stimulation intensity (10 to 50% of maximum output) with the custom-made Fig8 and Circ coils. There was a monotonic decrease in EMF for both Standard (and Reversed) and Sham TMS as the stimulation intensity decreased (Fig. 2B-2). For both the Fig8 and Circ coils, using one-way repeated measures ANOVA, EMF amplitude showed significant main effects of intensity (10, 30, 50%) for all TMS type (Standard/Reversed, Sham) and coils (Fig8, Circ) except for Sham TMS using the Fir8 coil (Fig8-Standard/Reversed: F_(2,57)_ =  = 10.1, p<0.001; Fig8-Sham: F_(2,57)_ = 1.42, p = 0.25; Circ-Standard/Reversed: F_(2,57)_ = 15652.4, p<0.001; Circ-Sham: F_(2,57)_ = 3002.9, p<0.001).

The results thus far show that EMF amplitude of Sham compared to Standard or Reversed TMS is significantly reduced. Further, Standard TMS and Reversed TMS have similar characteristics with the only difference being their polarities.

### Motor Physiology

In the third series of experiments, we performed motor physiological experiments to compare the levels of brain stimulation induced by Standard, Reversed and Sham TMS through the Fig8 and Circ coils. The coils were placed in an optimal orientation for Standard TMS (i.e., current flowing in the medial-anterior direction, which is in the perpendicular orientation to the central sulcus [20], [29]).

**Figure 3:**
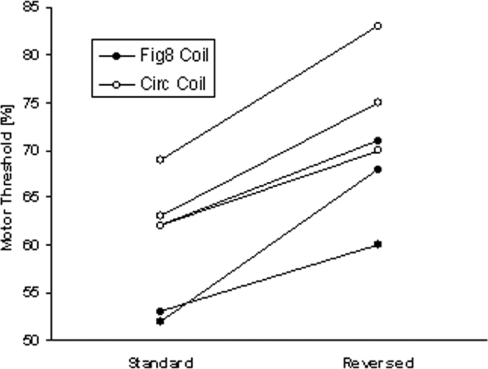
Motor Physiological Measurements. Subject motor thresholds are plotted for Standard and Reversed TMS through the Fig8 or Circ coil. Sham stimulation did not elicit muscle potentials even at maximum settings, thus threshold is beyond 100% stimulator output and not plotted.

Comparing Standard and Reversed TMS (Fig. 3), the motor threshold was higher for Reversed TMS (Fig8 coil: mean difference = 10.7, standard deviation (SD) = 4.7; Circ coil: mean difference = 11.3, SD = 3.1). This is consistent with the past literature indicating that when a coil is rotated by 180 degrees, that the motor threshold decreases by approximately 10.7% units of maximal stimulator output [21].

With Sham TMS, no MEPs could be detected even with maximal output, (and hence there was no measurable motor threshold) for neither the Fig8 nor the Circ coil.

### Perceptual Discrimination

In the next series of experiments, we tested whether naïve subjects and expert investigators could tell whether they received or applied Standard, Reversed or Sham TMS using the custom-made Fig8 and Circ coils. In order to simulate a realistic situation of a TMS experiment, naïve subjects performed a Stroop task (naming colors of words as accurately and as fast as possible where the words themselves were names of colors incongruent to the color of the words) while they discriminated between TMS types. While we intended this experiment for situations where single-trial TMS will be applied, this is not necessarily a realistic environment for some applications such as those intended for treatment, as subjects often do not perform any task while being stimulated. Experienced TMS researchers held the coil in their hand applying TMS and also attempted to discriminate between TMS types.

First, naïve subjects received 12 pulses of Standard, Reversed and Sham TMS and were then asked whether they had noticed different kinds of TMS pulses using the Fig8 coil. Since this was a debriefing experiment, we could only perform this test once for each subject.

None of the subjects were able to tell that there were different types of TMS intermixed using the Fig8 coil. When the subjects were specifically prompted to describe differences in strength or sensation from one pulse to another, none of the descriptions reflected the experimental manipulation. The following are sample impressions from subjects: ‘I didn’t notice anything different about the pulses… maybe intervals were random?’, ‘Did the intensity get stronger as the trials proceeded?’ (Intensity did not get stronger as trials proceeded), ‘I don't know, but I thought it switched sides, but only once.’ (TMS pulse did not switch sides).

**Figure 4:**
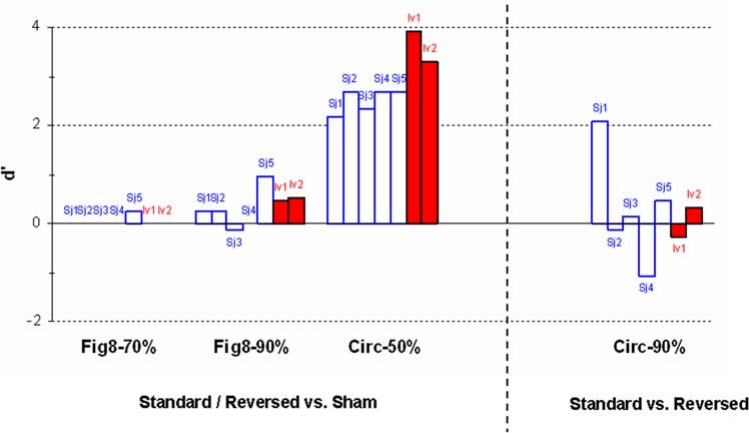
Perceptual Discrimination. Ability of five naive subjects (Sj1–Sj5; white bars) and two non-naïve investigators (Iv1, Iv2; red bars) to discriminate between stimulus conditions, expressed in terms of d' statistic (higher d' = greater discriminability). Discriminability between Real (Standard or Reversed) vs. Sham TMS through the Fig8 coil with stimulator at 70 and 90% of maximum stimulator output, and through the Circ coil at 50%. Also, discriminability between Standard and Reversed in the Circ coil with stimulator at 90%.

Prior to the next experiment, subjects went through a training period in which we administered several pulses of each type to serve as exemplars for Standard, Reversed and Sham TMS (approximately 5 pulses each). In the main experiment, subjects were asked to identify whether they received 1) a Standard or Reversed TMS, or 2) Sham TMS. As can be seen in Fig. 4, the d-prime (d', discriminability) values indicated that subjects could not tell whether they were receiving Standard/Reversed or Sham TMS with the Fig8 coil even when the stimulus intensity was set high at 70 or 90% (70%: mean d' = 0.05, SD = 0.12; 90%: mean d' = 0.27, SD = 0.42). However, all subjects could make the discrimination when a Circ coil was used, even at 50% of maximal output (mean d' = 2.51, SD = 0.25). These findings show that Standard/Reversed and Sham TMS could not be distinguished with a Fig8 coil and thus, the results of the Fig8 coil were promising as Sham TMS. Stacked Circ coils generate a lot of torque and sound in sham mode, and the field cancellation is nearly complete, even at the scalp hence producing little tactile sensation. We therefore expected that subjects will be able to discriminate Sham and Standard/Reversed TMS, but what was uncertain was whether subjects can correctly identify each mode. We hypothesized that if the subjects attended to the sound, then they would perceive Sham TMS as real TMS; alternatively if the subjects attended to the tactile sensation, then they would perceive Standard/Reversed TMS as real TMS. The findings of the Circ coil show that Standard/Reversed and Sham TMS could easily be distinguished.

Since Sham TMS using a Circ coil was easily distinguishable from Standard/Reversed TMS even at 50% of maximal output, we did not repeat the task at 70 or 90% maximal output. Instead, we investigated whether Standard TMS could be distinguished from Reversed TMS using the Circ coil. Reversed TMS could potentially serve as sham TMS since Reversed causes less cortical stimulation compared to Standard TMS when the coil is placed in an optimal orientation for Standard TMS (see Motor Physiology above and [21]). Results indicated that only one subject could distinguish between Standard and Reversed TMS using the Circ coil (mean d' = 0.30, SD = 1.14), raising the potential to use Reversed as sham TMS in the case of the Circ coil. While the number of subjects was small (N = 5), supplementary group statistics showed that conditions in which d' was significantly different from zero (d' = 0 indicates chance discrimination) was only when subjects discriminated between Standard/Reversed and Sham TMS applied with the Circ coil but not in other conditions including Standard vs. Reversed (t_(4)_ = 22.86, p<0.001; others t_(4)_ = 0.59–1.43, p = 0.23–0.59).

Overall Stroop performance was high (mean accuracy = 98.0%, SD = 2.2, range 92.7–100.0). There was no Stroop/TMS accuracy trade-off (i.e., there was no negative correlation between Stroop and TMS discrimination performance) indicating that the (in)detectability obtained was not dependent upon attentional load or difficulty of the concurrent task.

Expert non-naïve investigators holding the coil (as they might be when running TMS experiments) showed results similar to those of the naïve subjects above. They could not discriminate between Standard/Reversed and Sham TMS applied with the Fig8 coil or between Standard and Reversed TMS applied with the Circ coil, but could tell the difference between Standard/Reversed TMS and Sham TMS applied with the Circ coil.

#### Pain and Loudness Ratings

**Figure 5:**
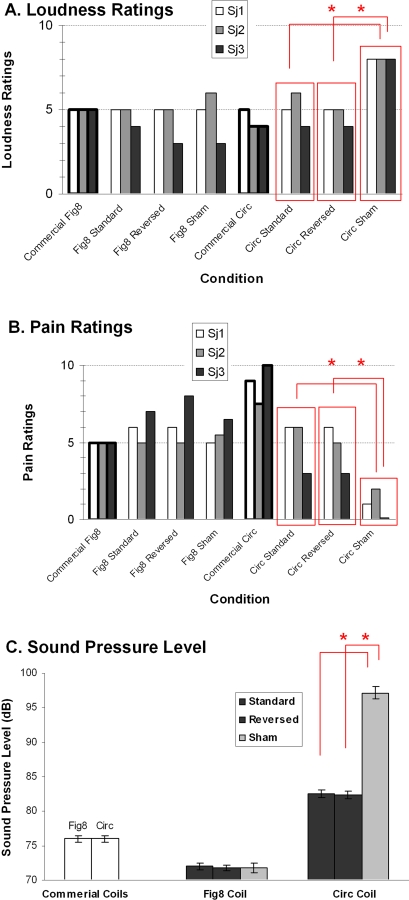
Loudness and Pain Sensation and Sound Levels. Subjective ratings for loudness (A) and for pain (B) from three naïve subjects for the three stimulus types. Stimulator was set at 90%. Ratings for stimuli from commercially available coils are included as reference. (C) Maximum sound pressure levels of the same stimuli. Error bars represent standard error of the mean. Comparisons with significant difference are marked with asterisks.

Subjects rated how painful and how loud TMS of the custom-made Fig8 and Circ coils were when applied to the prefrontal cortex, a typical site of stimulation in cognitive neuroscience research and clinical trials (Fig. 5; 10 trials/condition). For the Fig8 coil, no significant differences were found in either pain or loudness ratings in any subject for any of the pairwise comparisons between Standard, Reversed and Sham. (all p's >0.1). For the Circ coil, no significant differences were found in either pain or loudness ratings for any subject between Standard and Reversed (all p's >0.1) but there were (as expected) significant differences when ratings for Sham were compared to either Standard or Reversed; Sham was perceived as significantly louder (all p's<0.05) but also significantly less painful compared to Standard or Reversed (all p's<0.05). Discrimination of the Sham and Standard stimuli using the Circ coil in the Perceptual Discriminability Experiment was most likely due to these differences in tactile and auditory sensation. Pain and loudness ratings of commercially available Fig8 and Circ coils are shown in the figure as reference.

#### Sound Pressure Level (SPL) Measurements

Measurements of actual sound levels were not significantly different between Standard, Reversed and Sham TMS with the Fig8 coil (all p's >0.1). With the Circ coil, Sham TMS was significantly louder compared to the Standard and Reversed TMS (all p's<0.05). Sound levels of commercially available Fig8 and Circ coils are shown in the figure as reference.

## Discussion

In this study, we designed and validated a TMS stimulator attachment that allows for the administration of both Standard TMS stimulation and two types of control stimuli: Sham and Reversed. The spatial and temporal characteristics of the stimulation types were assessed by mathematical modeling and search coil measurements. The levels of brain activation elicited by the stimuli were assessed by measuring thresholds of activation in the motor cortex. The extent to which these stimuli could be interleaved in a double-blind manner was assessed directly in discrimination tasks, and indirectly via pain and loudness ratings and measurements of SPLs.

The electromagnetic and physiological effects of the equipment were found to be appropriate. Standard stimuli through the custom coils had profiles similar to the fields from ordinary commercial coils. Stimuli were consistent and unaffected by preceding stimuli of other types. Sham stimuli to motor cortex did not evoke MEPs even at 100% stimulator output. Reversed stimuli required significantly more power than Standard stimuli did in activating MEPs.

The perceptual experiments under realistic experimental conditions showed that naïve subjects and non-naïve investigators using the Fig8 coil in our experimental settings could not discriminate between the stimulus types. While individuals could easily discriminate between Sham and Standard stimuli delivered through the Circ coil (presumably due to Sham stimuli creating a loud sound and less tactile sensation), discriminability between Reversed and Standard stimuli was poor.

Stimuli with reversed polarity offer an alternative method of delivering control stimuli when an optimal direction of stimulation is known for the brain region of interest. Current-orientation-specific effects have not only been shown in the primary motor area [20], [29] but have also been reported in prefrontal and visual cortices [24], [25]. Because a change in stimulus polarity can change the threshold of activation in these regions, the effects of stimulation can be controlled without changing coil location or stimulus intensity. This is particularly meaningful for the use of the Circ coil, where Sham stimuli are discriminable. One should be careful when using reverse stimuli as a control condition, as reversal of the coil (which reduces the stimulation intensity by approximately 10–20 %) may not be sufficient in some cases and may also start recruiting undesired neuronal populations.

Another potential application of the equipment is its ability to stimulate opposite hemispheres of the brain without coil repositioning, with as little as 3 ms intervals. In the past, experimenters have found that flipping the coil over the same stimulation site can control the side on which lateralized motor and visual effects occur. The current switch offers such control with more stable coil positioning, and the ability to study inter-hemispheric interactions on a fairly short timescale.

In summary, we have developed and validated a TMS attachment that allows one to intermix real and sham TMS on a single-trial basis in a controlled double-blind manner. Experimental and control conditions can be alternated on a millisecond time-scale. Switching between conditions does not require coil movement or reconfiguration, and can be completely automated. The conditions can be double-blinded to counteract both placebo effects and experimenter bias. The attachment validated here is compatible with a commercially available TMS device in common use in the research community, and the simple design principle should be applicable to other TMS devices. Future research testing its perception when applied to other scalp sites, measurements of interindividual variability in larger number of subjects and application in ‘real’ research studies are warranted.

## Materials and Methods

### Subjects

A total of eight naïve and healthy subjects participated in the study (mean age 27.3, range 23–39, 2 females, 7 right handed). Two non-naïve healthy investigators who have been performing TMS experiments for at least the past 6 years (age 28, 36; 1 female; both right-handed) also participated in the study. None had contraindications to TMS [30], had known neurological or psychiatric disorders or were on medication. All subjects gave written consent and wore earplugs throughout the study. The study was approved by the Investigational Review Boards from California Institute of Technology and Stanford University School of Medicine.

### TMS Device

TMS was performed with two commercially available Magstim 200 stimulators and a Bistim module (Magstim Company, Carmarthenshire, UK). The inter-trial timing of the TMS pulses and the direction of current were controlled using Matlab (Mathworks, MA, USA) using Activewire (ActiveWire Inc., Campbell, CA, USA). Inter-stimulus-intervals (ISI) between two TMS pulses were controlled using the Bistim module when millisecond precision was necessary. Either commercially available Fig8 and Circ coils or the custom-made Fig8 and Circ coils were used. In case of the custom-made coils, the coils were connected through the custom switch-box attachment that allows switching between Standard, Reversed and Sham TMS. All data analyses were performed using Matlab. The custom setup was made to our specifications by Magstim in which we paid for labor and parts. Magstim had no intellectual input to the experimental design, interpretation of the results or in writing the manuscript.

### Mathematical Modeling of Electric Fields

The magnetic field 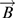 at a point r can be related to the current in the stimulating coil, I, by the law of Biot and Savart [31]

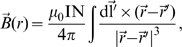
(1)where N is the number of turns in the coil, μ_0_ is the permeability of free space (4 π×10-7 T·m/A), and the integral of 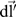 is over the coil path and 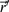 is a vector indicating the position of the coil path. The induced electric field can be calculated using the vector potential, 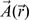, which is related to the current in the coil by the expression [31]

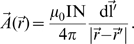
(2)The vector potential is in turn related to the electric and magnetic fields by the expression [31]

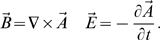
(3)


We calculated the integrals of Eq. (1) and (2) analytically along a line segment and then approximate our coil as a 32-sided polygon and summed the contribution from each side. Other assumptions made were following [32] and specifications of Magstim 200: capacitance (C) = 200×10^−6^ F, resistance (R) = 3 Ω, voltage of power source (V_0_) = 200 V, radius of wire = 1 mm, number of turns of coil (N) = 14, mean radius of coil = 4.5 cm for Circ and 3.5 cm for Fig8 coil, magnetic permeability (mu0) = 4 * pi * 1e-7, and measurement plane distance = 10 mm.

### Search Coil Measurements

Standard, Reversed and Sham TMS with the custom-made Fig8 and Circ coils and Standard TMS with commercially available Fig8 and Circ coils were applied to a search coil (one 10 mm diameter turn of copper wire) that was connected to an electric circuit. The search coil was placed at the center of the Fig8 coil where the two sets of coils intersect and on the turns of the Circ coil. This electric circuit was the same as that used in Corthout et al. [33]. The electric circuit consisted of a resistor R_1_ = 100 kΩ (representing longitudinal axonal resistance) in series with a parallel resistor R_m_ = 1 kΩ and capacitor C_m_ = 0.15 µF (representing membrane resistance and capacitance, respectively). Note that these values are not critical but were chosen to approximate the high longitudinal axonal resistance and a realistic membrane time constant [34]. The search coil EMF was recorded with an oscilloscope (Tektronix TDS 5104, Tektronix, OR USA).

Electro-motive force (EMF) was measured for single-pulse TMS and dual-pulse TMS with 10 ms ISI, single-pulse TMS while varying the distance of the search coil from the TMS coil surface, and single-pulse TMS while varying stimulus intensity. When dual-pulse TMS was applied, the order in which the two Magstim 200 stimulators were used to deliver TMS was pseudo-randomized and counter-balanced to avoid the effect of stimulator. There were 20 trials per condition. Statistical comparisons were performed using t-tests or one-way analysis of variance (ANOVA) on rise-time, maximum amplitude and area-under-the-curve of EMF. We report EMF amplitude as rise-time measures, area-under-the-curve measures showed similar results.

### Motor Physiology

Motor threshold was obtained for Standard, Reversed and Sham TMS using the Fig8 and Circ coils on three naïve subjects. First, subjects were seated comfortably and the coil was positioned using a coil-holder (Brainsight, Rogue Research Inc, Quebec Canada), at the scalp position at which Standard TMS induced motor-evoked potentials (MEPs) of maximal peak-to-peak amplitude in the target right first dorsal interosseus (FDI) muscle. Two electrodes were placed on the belly and tendon of the target muscle and a ground electrode of 30 mm diameter was placed on the right forearm after appropriate skin preparation. MEPs were collected using CED 1902 amplifiers (Cambridge Electronic Design (CED), Cambridge UK) with a band pass of 0.3–3,000 Hz. Following pre-amplification, the signal was digitized at a sampling rate of 6 kHz using a CED 1404 interface (CED, Cambridge, UK). Data was collected and analyzed using Signal software (CED, Cambridge, UK) and Matlab.

After the optimal location was determined and the coil positioned, motor threshold for Standard, Reversed and Sham TMS were determined for each subject in a pseudo-randomized and counterbalanced order. Motor threshold was defined as the minimal intensity of stimulation capable of inducing MEPs of more than 50 uV in at least six out of ten trials, during which the subjects maintained complete muscle relaxation, as documented by the electromyogram recording from at least 200 ms prior to TMS. There was an interval of 6–10 sec between trials. These were tested for both the Fig8 and Circ coils also in pseudo-randomized and counterbalanced order.

### Perceptual Discriminability

Five naïve subjects who were not part of the aforementioned motor physiology experiment performed a Stroop task while they performed perceptual discrimination tasks. We chose this study design because in most TMS studies, subjects perform a cognitive task while they receive TMS, and we wanted to test whether subjects can discriminate whether they received Standard or sham TMS applied to the top of the head (Cz according to the International 10–20 Electroencephalogram (EEG) system) under these conditions. Preliminary studies with TMS applied to the F3/F4 (prefrontal location) showed similar results. A list of color names was printed on the computer screen that was confirmed to be easily readable by subjects. The color of the printed words was incongruent to the names of the words. Subjects' were instructed to verbally name the color of the words as fast and as accurately as possible, and an investigator (AH) recorded all responses manually. Response times were not recorded as the detailed performance of the Stroop task was not the main focus of this study. Two non-naïve investigators also performed perceptual discrimination tasks while they held the coil in their hands. We chose to investigate non-naïve investigators applying TMS to test whether Standard/Reversed vs. Sham TMS can be blinded from investigators as well.

First, naïve subjects received 12 pulses of Standard, Reversed and Sham TMS at 90% maximum output and were asked at the end whether there were different kinds of TMS pulses using the Fig8 coil. We also asked them specifically whether they felt different intensities or sensations. Since questioning cued the subjects to the fact that there were three different types of TMS, we did not repeat this task using the Circ coil.

Next, we performed a two-alternative forced-choice task (2 AFC) using the Fig8 and Circ coils. Whenever TMS was applied to the subjects, in addition to continuing to perform the Stroop task, subjects were also instructed to press key 1 if they thought that they received real TMS (Standard or Reversed TMS) and 2 if they thought they received Sham TMS. There were a total of 40 pulses per task with 20 pulses or Standard or Reversed TMS and 20 pulses of Sham TMS. TMS was applied at a stimulus intensity of 50, 70 and 90% of maximal output for the Fig8 coil and 50% for the Circ coil. We did not test higher intensities for the Circ coil, as the subjects could easily identify whether they received a Standard/Reversed or Sham TMS.

Since the subjects could discriminate between Standard/Reversed TMS and Sham TMS for the Circ coil, we further tested to see if subjects could discriminate between Standard and Reversed TMS. There were 40 pulses of Standard and Reversed TMS (20 pulses each) at 90% stimulus intensity.

Hence, a total of 212 pulses were applied for the six tasks for each naïve subject: one debriefing task consisting of 12 trials, four tasks discriminating between Standard/Reversed and Sham TMS consisting of 40 trials per task, and one task discriminating between Standard and Reversed TMS consisting of 40 trials.

Two non-naïve investigators performed the 4 perceptual discrimination tasks while holding the TMS coil in their hands, without performing the Stroop task. Hence, there were 200 trials total per investigator.

To measure discriminability, d-prime (d') [35] was calculated for each subject using correct hits (correctly identifying Standard or Reversed TMS) and false alarms (incorrectly identifying Sham TMS as being Standard or Reversed TMS).

### Pain and Loudness Ratings

Three subjects who were part of the motor physiology experiment rated their perceptions of pain and acoustic intensity during TMS with Fig8 and Circ coils using a visual analogue scale (VAS; scale from 0 to 10, 0 being no pain/acoustic intensity, 5 being moderate pain/acoustic intensity and 10 being worst imaginable pain/loudest known acoustic intensity). These two measurements were obtained in separate experiments. TMS was applied at 90% maximal output to the left prefrontal region (F3 of the International 10–20 EEG system). This site was chosen because the prefrontal region is a common location to apply TMS in clinical trials of depression, as well as in cognitive neuroscience studies. Ten trials per condition (Standard, Reversed, Sham) were randomly intermixed, using either the Fig8 or Circ coil. Mean and standard error of the mean were calculated for each subject for each condition. Conditions were compared using paired t-tests between Standard and Reversed and between Standard and Sham TMS (p = 0.05).

### Sound Pressure Level Measurements

Maximum sound pressure levels of commercially available Fig8 and Circ coils as well as Standard, Reversed and Sham TMS using custom-made Fig8 and Circ coils at 90% maximum output were measured using a sound pressure monitor (A-weighted, 150 cm from coil surface; 10 trials per condition). Conditions were compared using paired t-tests between Standard and Reversed and between Real and Sham TMS (p = 0.05).

## Supporting Information

Text S1 Supporting Text
(0.05 MB DOC)

## References

[1] Hallett M (2000). Transcranial magnetic stimulation and the human brain.. Nature.

[2] Barker AT, Jalinous R, Freeston IL (1985). Non-invasive magnetic stimulation of human motor cortex.. Lancet.

[3] Hallett M, Chokroverty S (2005). Magnetic Stimulation in Clinical Neurophysiology..

[4] Chen R (2000). Studies of human motor physiology with transcranial magnetic stimulation.. Muscle Nerve Suppl.

[5] Maeda F, Pascual-Leone A (2003). Transcranial magnetic stimulation: studying motor neurophysiology of psychiatric disorders.. Psychopharmacology (Berl).

[6] George MS, Belmaker RH (2006). Transcranial Magnetic Stimulation in Clinical Psychiatry..

[7] Walsh V, Pascual-Leone A, Kosslyn SM (2005). Transcranial Magnetic Stimulation: A Neurochronometrics of Mind..

[8] Pascual-Leone A, Walsh V, Rothwell J (2000). Transcranial magnetic stimulation in cognitive neuroscience–virtual lesion, chronometry, and functional connectivity.. Curr Opin Neurobiol.

[9] Paus T (2005). Inferring causality in brain images: a perturbation approach.. Philos Trans R Soc Lond B Biol Sci.

[10] Lisanby SH (2004). Brain Stimulation in Psychiatric Treatment..

[11] Kobayashi M, Pascual-Leone A (2003). Transcranial magnetic stimulation in neurology.. Lancet Neurol.

[12] Okabe S, Ugawa Y, Kanazawa I (2003). 0.2-Hz repetitive transcranial magnetic stimulation has no add-on effects as compared to a realistic sham stimulation in Parkinson's disease.. Mov Disord.

[13] Vucic S, Kiernan MC (2006). Novel threshold tracking techniques suggest that cortical hyperexcitability is an early feature of motor neuron disease.. Brain.

[14] Jang SH, Cho SH, Kim YH, You SH, Kim SH (2005). Motor recovery mechanism of diffuse axonal injury: a combined study of transcranial magnetic stimulation and functional MRI.. Restor Neurol Neurosci.

[15] Mulleners WM, Chronicle EP, Vredeveld JW, Koehler PJ (2002). Visual cortex excitability in migraine before and after valproate prophylaxis: a pilot study using TMS.. Eur J Neurol.

[16] Robertson EM, Theoret H, Pascual-Leone A (2003). Studies in cognition: the problems solved and created by transcranial magnetic stimulation.. J Cogn Neurosci.

[17] Harrington A, Guess HA, Kleinman A, Kusek JW, Engel LW (2002). “Seeing” the placebo effect: historical legacies and present opportunities.. The Science of the Placebo.

[18] Schatzberg AF, Kraemer HC (2000). Use of placebo control groups in evaluating efficacy of treatment of unipolar major depression.. Biol Psychiatry.

[19] Kaptchuk TJ (2000). Debate over the history of placebos in medicine.. Altern Ther Health Med.

[20] Brasil-Neto JP, Cohen LG, Panizza M, Nilsson J, Roth BJ (1992). Optimal focal transcranial magnetic activation of the human motor cortex: effects of coil orientation, shape of the induced current pulse, and stimulus intensity.. J Clin Neurophysiol.

[21] Kammer T, Beck S, Thielscher A, Laubis-Herrmann U, Topka H (2001). Motor thresholds in humans: a transcranial magnetic stimulation study comparing different pulse waveforms, current directions and stimulator types.. Clin Neurophysiol.

[22] Pascual-Leone A, Cohen LG, Brasil-Neto JP, Hallett M (1994). Non-invasive differentiation of motor cortical representation of hand muscles by mapping of optimal current directions.. Electroencephalogr Clin Neurophysiol.

[23] Sakai K, Ugawa Y, Terao Y, Hanajima R, Furubayashi T (1997). Preferential activation of different I waves by transcranial magnetic stimulation with a figure-of-eight-shaped coil.. Exp Brain Res.

[24] Kammer T, Beck S, Erb M, Grodd W (2001). The influence of current direction on phosphene thresholds evoked by transcranial magnetic stimulation.. Clin Neurophysiol.

[25] Hill AC, Davey NJ, Kennard C (2000). Current orientation induced by magnetic stimulation influences a cognitive task.. Neuroreport.

[26] Ruohonen J, Ollikainen M, Nikouline V, Virtanen J, Ilmoniemi RJ (2000). Coil design for real and sham transcranial magnetic stimulation.. IEEE Trans Biomed Eng.

[27] Amassian VE, Eberle L, Maccabee PJ, Cracco RQ (1992). Modelling magnetic coil excitation of human cerebral cortex with a peripheral nerve immersed in a brain-shaped volume conductor: the significance of fiber bending in excitation.. Electroencephalogr Clin Neurophysiol.

[28] Illmoniemi RJ, Ruohonen J, Karhu J (1999). Transcranial magnetic stimulation-a new tool for functional imaging of the brain.. Crit Rev Biomed Eng.

[29] Mills KR, Boniface SJ, Schubert M (1992). Magnetic brain stimulation with a double coil: the importance of coil orientation.. Electroencephalogr Clin Neurophysiol.

[30] Wassermann EM (1998). Risk and safety of repetitive transcranial magnetic stimulation: report and suggested guidelines from the International Workshop on the Safety of Repetitive Transcranial Magnetic Stimulation, June 5–7, 1996.. Electroencephalogr Clin Neurophysiol.

[31] Jackson JD (1998). Classical electrodynamics..

[32] Roth BJ, Basser PJ (1990). A model of the stimulation of a nerve fiber by electromagnetic induction.. IEEE Trans Biomed Eng.

[33] Corthout E, Barker AT, Cowey A (2001). Transcranial magnetic stimulation. Which part of the current waveform causes the stimulation?. Exp Brain Res.

[34] Barker AT, Garnham CW, Freeston IL (1991). Magnetic nerve stimulation: the effect of waveform on efficiency, determination of neural membrane time constants and the measurement of stimulator output.. Electroencephalogr Clin Neurophysiol Suppl.

[35] Green D, Swets J (1966). Signal Detection Theory and Psychophysics..

