# Supplementary material for: Electronically Switchable Sham Transcranial Magnetic Stimulation (TMS) System
Source: PLoS One. 2008 Apr 9;3(4):e1923. doi: 10.1371/journal.pone.0001923 (PMC2271126; doi:10.1371/journal.pone.0001923)
Supplement: Text S1 — Supporting Text (0.05 MB DOC) [file pone.0001923.s001.doc]

**SUPPORTING INFORMATION**

**SUPPORTING INTRODUCTION**

Control stimuli can be generated either by redirecting the TMS field or by creating a null (sham) field. A survey of the methodologies used to generate such conditions reveals that the techniques currently employed in research cannot achieve double-blind interleaving of stimulus conditions, and introduce a number of experimental artifacts of their own.

Three techniques have been used to redirect the TMS field: 1) tilting the coil away from the scalp, 2) stimulating a control brain site, and 3) flipping the coil to reverse current polarity. The first two methods do not control for the physical sensations except to the extent that the subject feels scalp contact with the coil. Furthermore, it has been found, in some cases, that tilted coils still deliver effective levels of stimulation [1]. Similarly, when stimulating control brain sites, it is rarely known exactly what confounding effects might be involved. Finally, the coil movements required to switch between conditions make it difficult to keep stimulation sites stable.

The coil-flipping method is useful in instances where the brain region of interest has a lower threshold of activation for a particular polarity of stimulation (e.g., motor and visual cortices) [2-6]. By setting the magnitude of stimulation above the threshold for the optimal polarity and below threshold for the opposite polarity, the effectiveness of stimulation can be varied with a single site of stimulation. While the act of coil-flipping is problematic because of the movement and repositioning required, the resulting stimulation field is notable for being equivalent to the standard field in terms of absolute magnitude and distribution. Thus, it is possible that fields of reversed polarity might be used to vary the levels of brain activation while eliciting similar peripheral sensations. The capability to deliver reversed fields without coil movement is one of the techniques explored in this study.

For the administration of null-field stimulation, two categories of techniques have been employed: 1) simulation of peripheral sensations while coil remains inactive, and 2) use of separate placebo coils. In the first category, one group reproduced the coil click by discharging and extra TMS unit with its coil held nearby [7]. Another group combined this with electrical stimulation to provide scalp sensations [8]. These techniques are limited to inter-subject study designs, where control subjects never receive standard TMS.

In the second category, the only placebo coils on the market are made by Magstim (figure-of-eight coil; Wales UK) and by Neuronetics **(**figure-of-eight ferromagnetic core coil; Malvern PA USA). These coils provide slight sensory stimulation and discharge noise without stimulating cortical tissue. The sham coil by Magstim fails however, to truly mimic the peripheral sensations associated with TMS, and most subjects and researchers are able to distinguish between stimuli delivered from this placebo and from standard coils [9]. The sham coil by Neuronetics has not been validated. Such dedicated placebo coils share an additional disadvantage in that they have to be physically swapped with the standard coil. This poses the same problems of consistent localization noted earlier, and adds to it the inconvenience of swapping heavy cabling, (re-)clamping, etc. This not only makes interleaving conditions extremely inconvenient, but also heightens the subject’s awareness of the experimental manipulation.

A recent design for sham TMS, a “sandwich” configuration, involves stacking two standard TMS coils back-to-back, with a mu metal shield in between [9]. Standard and sham TMS are administered by discharging either the coil nearest the head or the coil behind the shield, respectively. This design is notable because it allows both stimulus types to be administered without switching or moving the coil. However subjects were found to be able to distinguish between the standard and sham TMS stimuli.

The design we present here builds upon a prototype by Ruohonen et al. [10]. This design also allows for the interchange of standard and sham stimulation without coil movement. They took a figure-of-eight coil design, and added a manual switch allowing them to reverse the current direction in one of the coil loops. This is in contrast to commercial figure-of-eight coils, in which current direction is fixed so as to be symmetric through the two loops, causing summation of the fields. Enabling the reversal of current in one of the loops allows for field cancellation, and thus sham stimulation. This design was implemented on a custom-built TMS setup and validated in terms of the motor and auditory potentials evoked by standard and sham stimuli.

**SUPPORTING REFERENCES**

1. Lisanby SH, Gutman D, Luber B, Schroeder C, Sackeim HA (2001) Sham TMS: intracerebral measurement of the induced electrical field and the induction of motor-evoked potentials. Biol Psychiatry 49: 460-463.

2. Brasil-Neto JP, Cohen LG, Panizza M, Nilsson J, Roth BJ, et al. (1992) Optimal focal transcranial magnetic activation of the human motor cortex: effects of coil orientation, shape of the induced current pulse, and stimulus intensity. J Clin Neurophysiol 9: 132-136.

3. Kammer T, Beck S, Thielscher A, Laubis-Herrmann U, Topka H (2001) Motor thresholds in humans: a transcranial magnetic stimulation study comparing different pulse waveforms, current directions and stimulator types. Clin Neurophysiol 112: 250-258.

4. Pascual-Leone A, Cohen LG, Brasil-Neto JP, Hallett M (1994) Non-invasive differentiation of motor cortical representation of hand muscles by mapping of optimal current directions. Electroencephalogr Clin Neurophysiol 93: 42-48.

5. Sakai K, Ugawa Y, Terao Y, Hanajima R, Furubayashi T, et al. (1997) Preferential activation of different I waves by transcranial magnetic stimulation with a figure-of-eight-shaped coil. Exp Brain Res 113: 24-32.

6. Kammer T, Beck S, Erb M, Grodd W (2001) The influence of current direction on phosphene thresholds evoked by transcranial magnetic stimulation. Clin Neurophysiol 112: 2015-2021.

7. Loo CK, Mitchell PB, Croker VM, Malhi GS, Wen W, et al. (2003) Double-blind controlled investigation of bilateral prefrontal transcranial magnetic stimulation for the treatment of resistant major depression. Psychol Med 33: 33-40.

8. Okabe S, Ugawa Y, Kanazawa I (2003) 0.2-Hz repetitive transcranial magnetic stimulation has no add-on effects as compared to a realistic sham stimulation in Parkinson's disease. Mov Disord 18: 382-388.

9. Sommer J, Jansen A, Drager B, Steinstrater O, Breitenstein C, et al. (2006) Transcranial magnetic stimulation--a sandwich coil design for a better sham. Clin Neurophysiol 117: 440-446.

10. Ruohonen J, Ollikainen M, Nikouline V, Virtanen J, Ilmoniemi RJ (2000) Coil design for real and sham transcranial magnetic stimulation. IEEE Trans Biomed Eng 47: 145-148.
